# Locating rearrangement events in a phylogeny based on highly fragmented assemblies

**DOI:** 10.1186/s12864-015-2294-6

**Published:** 2016-01-11

**Authors:** Chunfang Zheng, David Sankoff

**Affiliations:** Department of Mathematics and Statistics, University of Ottawa, 585 King Edward Avenue, Ottawa, K1N 6N5 Canada

**Keywords:** Genome rearrangements, Breakpoints, Phylogeny, Scaffold size

## Abstract

**Background:**

The inference of genome rearrangement operations requires complete genome assemblies as input data, since a rearrangement can involve an arbitrarily large proportion of one or more chromosomes. Most genome sequence projects, especially those on non-model organisms for which no physical map exists, produce very fragmented assembles, so that a rearranged fragment may be impossible to identify because its two endpoints are on different scaffolds. However, breakpoints are easily identified, as long as they do not coincide with scaffold ends. For the phylogenetic context, in comparing a fragmented assembly with a number of complete assemblies, certain combinatorial constraints on breakpoints can be derived. We ask to what extent we can use breakpoint data between a fragmented genome and a number of complete genomes to recover all the arrangements in a phylogeny.

**Results:**

We simulate genomic evolution via chromosomal inversion, fragmenting one of the genomes into a large number of scaffolds to represent the incompleteness of assembly. We identify all the breakpoints between this genome and the remainder. We devise an algorithm which takes these breakpoints into account in trying to determine on which branch of the phylogeny a rearrangement event occurred. We present an analysis of the dependence of recovery rates on scaffold size and rearrangement rate, and show that the true tree, the one on which the rearrangement simulation was performed, tends to be most parsimonious in estimating the number of true events inferred.

**Conclusions:**

It is somewhat surprising that the breakpoints identified just between the fragmented genome and each of the others suffice to recover most of the rearrangements produced by the simulations. This holds even in parts of the phylogeny disjoint from the lineage of the fragmented genome.

## Background

The study of genome rearrangements generally requires a relatively complete assembled genome, consisting of a few lengthy scaffolds made up primarily of long contigs, preferably anchored to a physical map. In practice, however, *de novo* sequences of non-model organisms will consist of many short scaffolds, most containing no genes, and with no direct information on how to order the scaffolds with respect to each other [1].

Algorithmic inference of rearrangements [2] requires the possibility of identifying chromosomal fragments that have been inverted, inserted, deleted, duplicated, transposed to a new location on the chromosome or translocated from one chromosome to another. This involves identifying the whole fragment, including the two endpoints, which are in different genomic contexts before and after the rearrangement operation, and hence define one or more breakpoints. Most such fragments cannot be detected in a short-scaffold assembly, since only the shortest of fragments generally will fit on a single scaffold.

Nevertheless, much can be learned from identifying single breakpoints, since each results from a rearrangement event, and conversely each identifiable rearrangement event gives rise to one or more breakpoints, so that the number of rearrangements is somewhat less than the number of breakpoints, depending on the evolutionary processes at play [3].

In this paper, we consider how to use breakpoint comparisons in a phylogenetic context, not by computing a distance matrix among genomes, but in locating rearrangement events on the branches of a phylogenetic tree. Prior to the eventual implementation of any practical tool for this task, we focus solely on the effect of fragmentation of just one of the genomes in the phylogeny on the integrity of rearrangement inference, with a range of degrees of fragmentation and various amounts of rearrangement. To isolate this effect, in our simulations, we will deliberately simplify the context to rearrangement by random inversion of unichromosomal genomes with the same, single-copy, gene content. And we will constrain ourselves to comparisons of the one fragmented genome in a phylogeny to all the other intact genomes.

In the course of our exposition, and especially in the conclusions, we will refer to how this simplified model would have to be amended in dealing with real genomes. Nevertheless, the findings of the present model with respect to degree of fragmentation, rate of rearrangement and phylogenetic accuracy should be pertinent to more realistic data.

## Methods

### From the unambiguity of gene adjacencies to the indeterminacy of block adjacency

In gene order comparisons, it is necessary to work with blocks of genes conserved in two or more genomes; trying to work with one gene at a time is not a robust procedure, especially with flowering plants, because most of these genomes have a whole genome duplication (WGD) in their history. The fractionation process ensuing from WGD deletes duplicate genes in a partially random pattern from one or the other duplicate (homeologous) chromosome, independently in two or more descendants of a duplicated genome [4]. This pattern, together with the possibility for some genes to transpose into different positions in the genome, makes it hard to identify unambiguously orthologous genes that are in the same gene order in two genomes. A set of five or ten genes in the same order, with few intervening genes, in two genomes can be confidently identified as a conserved syntenic block [5, 6].

However, the notion of block adjacency encounters a number of operational problems; the genes in a syntenic block in one genome may differ somewhat from the same block in the other genome, the minimum number of genes to establish a block is a parameter that must be determined by some empirical experimentation, as is the number of genes allowed to intervene between two pairs of orthologs within a block in the two genomes. We will avoid these practical problems in our simulations by excluding fractionation or other gene loss, duplication and small transpositions from our model.

## The principle of event assignment

By assuming no evolutionary coincidences in rearrangement between large genomes, we derive the following principle.

Any breakpoint observed in two genomes must have occurred on the evolutionary path through the phylogeny leading from one of the genomes to another. Conversely, if there is an adjacency in common between the two genomes, there can have been no rearrangements affecting the corresponding scaffolds containing this adjacency in the two genomes apparent anywhere on the path between them.

In observing the distribution in a known phylogeny of which genomes conserve a given adjacency and in which it is disrupted, this principle suffices to infer the identity of the branch or a small subset of the branches where the rearrangement causing this adjacency occurred.

If a genomic comparison violates this principle, we say there is a conflict. In general a conflict will become apparent as by the appearance of a vertex reconstructed somewhere on a path between two genomes with no breakpoint.

These principles hold even if some of pairs of genomes are “can’t tell” as to the whether they have a certain adjacency in common, and whether or not we are dealing with a binary or multifurcating phylogeny. In these cases, inference cannot necessarily locate a single branch where an event must have occurred, but only identify a subset of such branches.

## The algorithm for the general case (with “can’t tell” vertices)

We consider all rooted or unrooted trees *T*, binary or not, with leaves named 1,…,*N*. Given a set of fragmented genomes {*g*_1_,…,*g*_*N*_}, we designate one at a time, say *g*_*i*_ as the reference genome. For each scaffold *s*∈*g*_*i*_, we label each other genome *g*_*j*_ as 1,2, or 3 depending on whether the content of *s* is “split”, according to some formal criterion as described above, among two or more scaffolds of *g*_*j*_, is entirely contained in one genome of *g*_*j*_, or is “can’t tell”. We then run the following dynamic programming algorithm.

- Notation:

- unrooted tree *T*=(*V*,*E*)
- *T*_*v*_: a subtree subtended by vertex *v*
- possible vertex labels: {1 (split), 2 (not split), 3 (“can’t tell”) }
- event: edge with one vertex labelled 1, the other labelled 2.
- eventNumber (*v*): number of events in *T*_*v*_:

- Input:
- unrooted tree *T*
- leaf vertices labelled 1, 2, or 3.

- Output:
- *S*, a smallest possible set of event edges.

Algorithm: to label each non-leaf vertex with label 1 or 2, carry out a standard dynamic programming procedure [7] using labels 1, 2 and 3. During the traceback, replace every label 3 by a 1 or a 2, and consider the set of all possible results. This determines the set of branches on which an event could have occurred, namely the set of branches which in some solution are labeled 1 at one end and 2 at the other.

## Results

All the experiments reported here were carried out on randomly generated binary unrooted trees based on seven input genomes, each having four internal branches, and all results are averages of a sample size of 100 simulations.

To produce each data sample, one of the internal nodes is chosen as a root genome. It is defined to be a single chromosome containing 1000 genes. The rearrangements we apply are inversions with two randomly chosen endpoints. (The choice of root is immaterial because of the symmetry of the inversion operation). After each simulation run, a designated genome is randomly split into a certain number of scaffolds, whose sizes are approximately geometrically distributed. Note that this is the simplest possibly case; in general, more or all of the genomes could be fragmented.

## The effect of evolutionary time

**Fig. 1:**
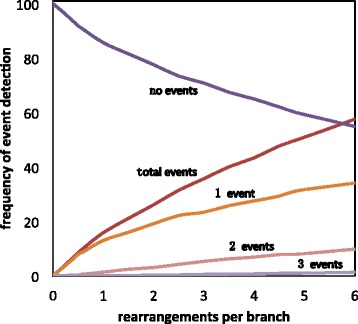
Events detected as a function of amount of rearrangement. Designated genome fragmented into 100 scaffolds. “2 events” or “3 events” count rearrangements affecting a single scaffold in the designated genome. Total events = number of “1 events” + 2 × number of “2 events” + 3 × number of “3 events”

In the first experiment, Fig. 1 shows that as expected, an increasing number of genome rearrangements is reflected in an increasing number of breakpoints detected. In these simulations, one rearrangement generally produces two breakpoints. With larger numbers of rearrangements, sometimes only one new breakpoint is created, the other being “re-used”. When there are several branches with events in a lineage, we can usually only detect that at least one occurred.

The results of the — experiment show that, for example, with one rearrangement per branch almost 20 breakpoint events are detected on our 11-branch (7 terminal and 4 internal branches) tree but that many scaffolds do not contain a breakpoint, and some scaffolds contain several, which cannot easily be teased apart.

## The effect of poor assembly

**Fig. 2:**
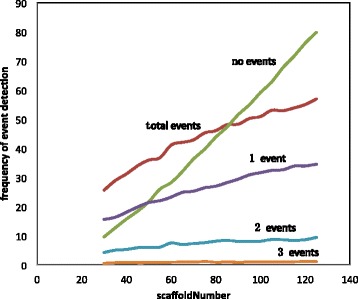
Events detected as a function of amount of fragmentation. Simulations involved 5 rearrangements per branch. Increasing the number of scaffolds reduces the chance breakpoints from two rearrangements will be on a single scaffold and thus counted as a single event. It also sharply increases the number of scaffolds containing no breakpoints

Figure 2 shows the results of our second experiment where increasing the number of scaffolds leads to an increased probability that a given scaffold will contain no breakpoints. But it also decreases the probability that several rearrangements will involve the same scaffold.

## Phylogeny based on the fragmented genome

The preceding experiments illustrate the use of the comparative, phylogenetic context to date most of the rearrangement events on the branch of a tree, or possibly a small subset of branches. Perhaps the main application of our method will be to actually distinguish among candidate trees, solely on the basis of rearrangements affecting the designated genome. Thus we run the algorithm for all 945 binary unrooted trees on seven genomes, not just the one *T* we used to generate our data, and check whether *T* is the most parsimonious with respect to the number of inferred events. Note that this experiment coincides with choosing the phylogeny with the smallest number of conflicts in pinpointing the rearrangement events.

**Fig. 3:**
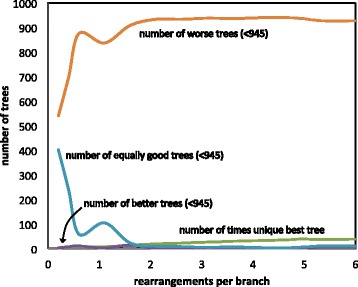
Phylogenetic accuracy based on event detection. Designated genome broken into 60 scaffolds. Data-generating tree *T* is among the best 10 or 12, out of 945, in almost all the simulations

In Fig. 3, we see that with even a small number of rearrangements per branch the tree *T* that generated the data is the best or among the 1 % best trees. In general, the trees with an equivalent or better score will be very similar in topology to *T*.

## The effect of unidentifiable breakpoints

**Fig. 4:**
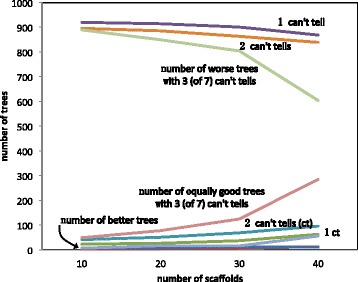
Effect of “can’t tells” on phylogenetic accuracy. With 3 out of 7 genomes exhibiting a “can’t tell”, the method becomes sensitive to the number of scaffolds

With real data, especially in the context of intense genomic fractionation, such as with the flowering plants, there will be a high incidence of “can’t tell”. By randomly re-identifying some of identified breakpoints as “can’t tell”, we can see the effect of degrading the data to model these real cases. Figure 4 shows that the method is stable to low levels of “can’t tell”, but tends to break down with increasing fragmentation when almost half the genomes exhibit “can’t tell” with a designated genome scaffold.

## Discussion

Our simulations were set up in as simple a way as possible to explore the main potentials and the limitations of event detection based on observed breakpoints involving a single fragmented genome, the rest being completely assembled. We have studied the effects of the degree of fragmentation (number of scaffolds), rearrangement rate (number of inversions) and the rate of “can’t tells”. There would be many other secondary parameters to investigate in more general experiments: changing the number of genomes, allowing more or all of them to be fragmented, having larger and more heterogeneous genome sizes, and incorporating translocations, transposition, genome duplication and other rearrangement operations into a more variable and realistic model of gene order evolution, rather than the simple random inversion. Most important, confining our analysis to the phylogenetic viewpoint from a single genome is responsible for much or most of the shortfall in breakpoints and would be greatly attenuated in an analysis combining the viewpoints of all the genomes.

The most important difficulty in empirical work on this topic is determining the location and identity of breakpoints and determining if two breakpoints, on two genomes other than the designated one, are in fact the same or different. This difficulty stems from three sources, the first being fractionation, which removes gene copies more or less at random, so that single gene absences produce an apparent breakpoint; this necessitates examining neighboring syntenic blocks of genes, rather than single genes, to determine adjacencies and breakpoints, involving a whole new set of methodological problems.

The second source is the presence of numerous small, local, species-specific rearrangements, which we may or may not want to consider in a phylogenetic study spanning tens of millions of years of gene order evolution. Again, syntenic block comparisons help attenuate this problem.

Third, duplication of segments and whole genome duplications can complicate the determination.

On the theoretical level, the main interesting extensions would be the treatment of “can’t tells” and of non-binary trees. Here the non-uniqueness is due not only to the distinct, conflicting, solutions occurring in special data configurations discussed above, but more generally in solutions where an event is identified as occurring somewhere on a path or other subtree, rather than on a specific branch. For phylogenetic scoring, this can be handled by splitting the unit score for the scaffold in question into fractions to redistributed among all the alternate branches.

There is a conceptual similarity between the problems we have studied here and the strategy of using comparative data to assemble contigs into scaffolds [8, 9]. In practice, however, the level of noise simulated in the current work, is greater than that in the data used for “contig fusion” or “scaffold filling”, where minimizing genome rearrangement is a strategy for optimizing contig and scaffold orders.
